# Oxidative Stress and the Pathogenesis of Aortic Aneurysms

**DOI:** 10.3390/biomedicines12010003

**Published:** 2023-12-19

**Authors:** Matthew Kazaleh, Rachel Gioscia-Ryan, Gorav Ailawadi, Morgan Salmon

**Affiliations:** 1 Department of Cardiac Surgery, Michigan Medicine, University of Michigan, Ann Arbor, MI 48109, USA; mkazaleh@med.umich.edu (M.K.); ailawadi@med.umich.edu (G.A.); 2 Department of Anesthesiology, Michigan Medicine, University of Michigan, Ann Arbor, MI 48109, USA; rgioscia@med.umich.edu; 3 Frankel Cardiovascular Center, Michigan Medicine, University of Michigan, Ann Arbor, MI 48109, USA

**Keywords:** reactive oxygen species, aortic aneurysm, abdominal aortic aneurysm, thoracic aortic aneurysm, oxidative stress, antioxidant

## Abstract

Aortic aneurysms are responsible for significant morbidity and mortality. Despite their clinical significance, there remain critical knowledge gaps in the pathogenesis of aneurysm disease and the mechanisms involved in aortic rupture. Recent studies have drawn attention to the role of reactive oxygen species (ROS) and their down-stream effectors in chronic cardiovascular diseases and specifically in the pathogenesis of aortic aneurysm formation. This review will discuss current mechanisms of ROS in mediating aortic aneurysms, the failure of endogenous antioxidant systems in chronic vascular diseases, and their relation to the development of aortic aneurysms.

## 1. Introduction

Aortic aneurysms (AAs), defined as a progressive and pathologic dilation of the aorta, are responsible for 150,000–200,000 deaths per year worldwide [1]. Despite this clinical significance, there remain critical knowledge gaps in the mechanism and pathogenesis of all types of AAs, and how these differences can increase rupture potential. Current understanding of the pathogenesis of sporadic AAs suggests a complex interplay of protein degeneration, thrombosis, hemodynamic stress, and inflammatory cytokines [2]. Recent studies by our lab and others have drawn attention to the role of reactive oxygen species (ROS), antioxidant pathways such as itaconate and other TCA derivatives, and the down-stream effects of these pathways in AA pathogenesis and rupture [3,4]. Emerging evidence suggests ROS pathways modulate lipid homeostasis, protein stability and location, epigenetics, mitochondrial fission and fusion, and energy metabolism and when present in relative excess, such as a state of oxidative stress, drive pathophysiologic changes in cellular signaling. Evidence also supports a role for ROS in the regulation of chronic inflammation, including in the setting of vascular diseases such as atherosclerosis and aortic aneurysms [1,5,6]. Therefore, understanding the ability of ROS to influence the signaling pathways underlying AA formation or rupture and may provide novel insight for routes of clinical intervention. This review will evaluate the link between known mechanisms of inflammation and potential roles of ROS in the activation of these inflammatory pathways in AAs. We will also discuss the role of oxidative stress, whether due to excessive ROS production or deficiency of endogenous antioxidant defenses, in AAs. Finally, we highlight endogenous antioxidant defense systems as potential therapeutic targets in aortic aneurysm disease.

## 2. Aortic Aneurysms

AAs are characterized by at least 50% increased dilation of the aorta [7]. Categorized based on their anatomic location, AAs are generally divided into three distinct types; ascending aortic aneurysms (aAAs) located in the aortic arch, descending thoracic aortic aneurysms (dTAAs) located proximal to the diaphragm in the thoracic aortic cavity, and abdominal aortic aneurysms (AAAs) located between the infrarenal abdominal aorta. AAs possess unique genetic and/or environmental risk factors with varying levels of penetrance based on location. For example, mutations in genes such as *fibrillin, ACTA2, MYH11* and *LOX* have been found to influence aneurysms of the ascending aorta [8]. Conversely, AAAs tend to develop with aging and in conjunction with certain risk factors such as gender, tobacco use, and cardiovascular disease. A crucial shared characteristic of all types of AAs is the elevated morbidity and mortality associated with aortic rupture. All types of AAs are generally clinically silent until impending dissection and/or rupture, and when rupture does occur, mortality approaches 90% [8,9]. Therefore, the development of a medical treatment therapy to prevent aortic rupture would address a significant medical health care burden.

While all AAs involve progressive dilation of the aorta, the mechanisms of the localized dilation and capacity to rupture are different based on the anatomical location and embryological origin of the resident cells. The lack of comprehensive knowledge of the causal mechanisms of AAs and effective strategies for intervention is reflected in the growing incidence of AAs. In 2022, the prevalence of AAA was an estimated 0.92%, representing roughly 35 million individuals [10].

It is commonly believed that AA formation and rupture are multifactorial processes that involve a combination of genetic and environmental factors, both known and unknown, to produce localized aortic dilation. The first known key difference between AAs in the thoracic vs. abdominal aorta stem from the unique cellular and structural composition of the residential architecture of the thoracic and abdominal aorta. The ascending and descending thoracic aorta exhibit thinner intima, thicker media, and a higher elastin and collagen content when compared to the abdominal aorta [11]. These differences have recently been attributed to the embryological origin of the resident cells as vascular smooth muscle cells (VSMCs) of the thoracic aorta are of neural crest origin, whereas abdominal VSMCs originate from mesoderm and endothelial cells [11,12,13]. However, in both cases the dysregulation of VSMC function has been linked to the formation and progression of AA [14]. The unique segmental composition of the thoracic and abdominal aorta provides the framework for pathogenetic mechanisms to impart effects of unequal magnitude and leads to differential AA characteristics based on anatomical location. Recent evidence suggests that TAAs are fibrotic diseases of modulated VSMCs and fibroblasts while AAA pathology is reflective of a chronic inflammatory disease of the aging aorta. However, it is important to note that there remain key unresolved knowledge gaps in the pathogenesis of both types of AA disease that prevent regional-specific medical treatment therapies from being developed.

Another key difference in thoracic and abdominal aortic aneurysm formation is the genetic predisposition to aneurysm formation based upon location. Approximately 30% of TAA cases have a genetic component from clinical syndromes or connective tissue disorders such as Marfan, Loeys-Dietz, and Ehlers–Danlos Syndrome, associated with mutations in genes such as *fibrillin*, *ACTA2*, *MYH11* and *LOX* [8]. In contrast, there has not been a strong genetic link to AAAs.

Both TAA and AAA share causal environmental risk factors such as tobacco use, hypertension, atherosclerosis, age, and male sex [15]. Risk factor modification remains the only means of medical management of AAs, leaving a significant need for the development of novel medical treatment therapies. Current treatment strategies include invasive open surgical or endovascular repair when the aorta reaches a crucial size or enlarges at a rapid rate; however, these procedures are associated with substantial morbidity and represent a significant medical treatment burden to current health care systems [16,17,18]. Size threshold and growth rate are currently key triggers for intervention, and this intervention does not come without risk. Risk factors such as increased age and female gender have been associated with higher mortality rates following surgical intervention [18]. Given the morbidities of aortic intervention, a significant effort to establish alternative means of medical management is underway. There exists a window of potential medical intervention between diagnosis and invasive surgical treatments in which targeted medical therapy could halt progression or prevent aortic aneurysm rupture.

## 3. Inflammatory Mediators of Aortic Aneurysm Formation

As previously discussed, the pathogenesis of aortic aneurysm formation is both complex and multifactorial. Generalized destruction of elastin and collagen in the aortic media and adventitia, VSMC loss, and the infiltration of lymphocytes and macrophages are all pathophysiologic mechanisms that propagate aneurysm formation [11]. Dysregulation of the physiologic process of inflammation is considered a key contributing upstream mechanism of AA formation. Activated macrophage and pro-inflammatory CD4 T-cell infiltration has been linked to aneurysm formation and rupture in murine models [11,19]. This process is driven by several inflammatory mediators which have been linked to cardiovascular diseases, including IL-1β, the NLRP3 inflammasome, NF-Kβ, and IL-6. We aim to introduce the intricate connection among dysregulation of these inflammatory mediators, ROS signaling, and AA formation.

**Figure 1:**
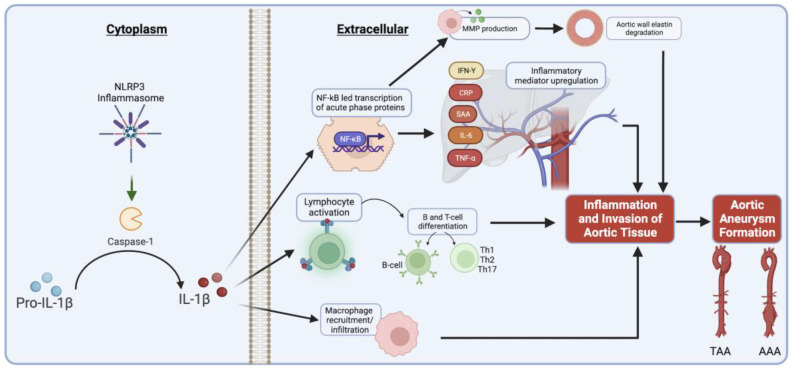
IL-1β activation via caspase-1, with associated extracellular inflammatory mediator upregulation (figure generated via biorender.com, accessed 1 November 2023).

In the setting of aortic aneurysm formation, IL-1β serves as a potent inflammatory cytokine and acts primarily through its receptor, IL-1R1 [16]. Recent evidence from our lab and others suggests that IL-1β and IL-1α, which both bind the IL-1R1 and in some cases are believed to have overlapping functions, are not functionally redundant and that IL-1α could exert protective effects in AAs. In contrast, IL-1β has various downstream inflammatory effects, perhaps its most well-known being stimulation of macrophage infiltration into the aortic wall during AA formation [17]. IL-1β activation occurs primarily through cleavage of its precursor via the NLRP3 inflammasome, which also results in the increased activity of the pro-inflammatory caspase-1 [16,17,20]. This protease cleaves pre-IL-1β into its active form, which is released extracellularly and binds to effector cells and lymphocytes via IL-R1, propagating further inflammatory mediators such as TNF-α, IL-6, MCP1 and IFN-Y (Figure 1) [19,21]. Previous studies have identified a 20-fold increased level of IL-1β in human TAA samples when compared to non-aneurysmal human aortic samples [17]. Conversely, genetic deletion of IL-1β in murine models has been found to preserve aortic elastin and smooth muscle cells, with less pronounced inflammatory cell infiltration, and attenuation of AAA and dTAA formation [17,20]. Additionally, deficiency of CD4 Th17 cell signaling, a process driven by IL-1β, is associated with reduced aortic macrophage infiltration and attenuated aneurysm formation in murine models [21]. Canakinumab, a monoclonal antibody to human IL-1β, has been shown to suppress tumorigenic inflammatory pathways and angiogenesis in certain types of lung cancers, and is currently under investigation for effects in cardiovascular disease [22,23]. These studies establish the role of IL-1β as an early mediator of AAs.

As previously mentioned, the assembly and activation of the NLRP3 inflammasome, a multiprotein complex, results in caspase 1 activation and the dependent release of IL-1β, with associated increases in cytokines such as IL-18 [24,25,26,27]. The NLRP3 inflammasome represents a critical upstream mediator of pro-inflammatory cytokines and is therefore a potential “gatekeeper” for pro-inflammatory states and diseases such as AAs. Studies in NLRP3 knockout murine models have shown reduced aortic destruction and aneurysm formation, suggesting the NLRP3-caspase-1 inflammasome is contributory in aortic aneurysm formation [28]. Additional investigation in Apolipoprotein E (ApoE) –/– mice (deficient in NLRP3, caspase recruitment domains, and caspase-1) found reduced elastic lamina degradation and MMP activation in early AAA formation, further supporting a role for the NLRP3 inflammasome in aneurysmal disease [29]. Some current research efforts have investigated the potential effects of NLRP3 inflammasome inhibition through the use of pharmacologic inhibition, representing a possible avenue for clinical intervention in AAs, especially given the mixed effectiveness of the IL-1 pathway in other chronic vascular diseases [30,31].

NF-Kβ is a protein complex that acts as a cytokine-responsive transcription factor, stimulating the production of matrix metalloproteinases (MMP) in macrophages and other acute phase inflammatory reactants and is a known down-stream effector of IL-1β signaling. The intricate NF-Kβ cascade contributes to the breakdown of aortic wall integrity during the formation of AAs [8]. Elevated levels of NF-Kβ have been detected in human thoracic (TAA) and abdominal (AAA) aortic tissues when compared to non-aneurysmal control tissues, associated with increases in MMP-2 and MMP-9 [32]. In murine models, the inhibition of NF-Kβ has been demonstrated to attenuate AAA formation through a reduction in endothelial adhesion molecule expression, which is thought to trigger macrophage infiltration and inflammatory sequela in the aortic adventitia and media [33,34]. IL-6 is a downstream chronic and acute phase inflammatory cytokine with a key role in aortic aneurysm formation, involved in stimulation of protein synthesis, neutrophil production, and macrophage recruitment [35]. As previously discussed, activation of secondary inflammatory mediator IL-6 is driven by IL-1β activation, through a cascade activation of mitogen-activated protein kinases [36]. IL-6 has been linked to chemokine and chemokine receptor-mediated inflammatory cell migration in the setting of AAA [30]. Levels of circulating IL-6 have been found elevated in patients with AAA and TAA and correlated with the size of the aneurysm in cross-sectional studies [37,38]. Murine models have established that IL-6-knockout mice exhibit TAA attenuation, suggesting a possible novel target for aneurysm prevention in humans [39].

Although the role of inflammatory signaling in aortic aneurysm development is well investigated, the precise cellular processes and mechanisms underlying increased inflammation in aneurysmal disease remains incompletely understood, especially in chronic growth and rupture. A growing body of evidence suggests that oxidative stress, characterized by an excess of ROS production relative to endogenous antioxidant defenses, can incite pro-inflammatory signaling and potentially play a substantial role in the progression of vascular diseases, including AAs.

## 4. Reactive Oxygen Species Link to Aortic Aneurysms

ROS are chemically reactive molecules that contain at least one oxygen molecule and one or more unpaired electrons that are derived from redox reactions and are formed as a natural by-product of aerobic metabolism [3]. Common species of ROS include superoxide anion (O_2_^−^), hydroxyl radical (^−^OH), hydrogen peroxide (H_2_O_2_), nitrous oxide (N_2_O), peroxynitrite (ONOO^−^), and hypochlorite (ClO^−^). These molecules possess physiologic roles when produced appropriately and in limited quantities, including but not limited to the modulation of cell survival/death, immunologic differentiation, and modulation of physiological functions [40]. Many ROS-mediated responses protect cells against oxidative stress and reestablish redox homeostasis [41]. As the mitochondria perform the majority of cellular metabolism, they are an important source of ROS and generate approximately 90% of cellular ROS in certain tissue types [42]. During periods of oxidative stress, the over-production of ROS leads to imbalance, resulting in the oxidative damage of cellular components [42]. Oxidative imbalance results in pathophysiologic consequences, and the recent literature suggests increased oxidative stress alters signaling pathways responsible for inflammation and pathogenesis of cardiovascular diseases [2]. Atherosclerosis, hypertension, cardiomyopathy, cardiac arrhythmias, and AAs have all been linked to redox imbalance [40,41,42]. Of specific relevance to AAs, overproduction of vascular ROS results in increased MMP activity, vascular smooth muscle cell apoptosis, and alteration to aortic wall collagen integrity [6,43].

The current literature has established multiple pathways contributing to the generation of ROS in AAs. Notably, pathways associated with NADPH oxidase and iNOS are responsible for the generation of the superoxide anion (O_2_^−^), a factor consistently associated with AAA formation in numerous studies [43,44,45]. Pioneering research by Xiong et al. determined that the selective genetic knockout of iNOS as well as NADPH oxidase inhibition with orally administered apocynin resulted in the decreased expression of MMP-2 and MMP-9, lower levels of NO_2_ and NO_3_ production, and a notable reduction in AAA formation [45].

Another important redox pathway in vascular physiology and AA disease is endothelial nitric oxide synthase (eNOS). This enzyme serves a pivotal role in safeguarding vascular cells against oxidative harm. By generating nitric oxide (NO^−^), it deactivates superoxide anions and other ROS, thereby serving as a protective mechanism against deleterious oxidative stress. Nitric oxide, when present at appropriate levels, acts as a potent vasodilator necessary for maintaining hemodynamic balance and is also critical for promotion of a cellular milieu which favors vasodilation and inhibition of inflammation, proliferation, and coagulation. Research has demonstrated that suboptimal nitric oxide levels or limited bioavailability contribute to the worsening of atherosclerosis [46]. In cases of advanced atherosclerosis, damage to the vascular endothelium due to lipid plaque deposition disrupts laminar blood flow, triggering the upregulation of inflammatory pathways. This oxidative stress perpetuates a positive feedback loop, exacerbating both local and systemic inflammation. When eNOS loses its connection with vital cofactors like tetrahydrobiopterin, it leads to the formation of superoxide anion (O_2_^−^) instead of nitric oxide (NO^−^), resulting in endothelial dysfunction and vascular disease. Siu et al. confirmed in murine models of AA disease the consequences of uncoupled eNOS [47]. They found uncoupling eNOS with vital cofactor tetrahydrobiopterin (H_4_B) led to the development of abdominal aortic aneurysms (AAA) in hph-1 mice [47]. Interestingly, supplementation with folic acid, known to reestablish the coupling of eNOS by enhancing dihydrofolate reductase (DHFR) function, significantly reduced AAA formation in these mice [48,49].

As previously mentioned, mitochondria are a source of ROS and account for approximately 90% of total cellular ROS in some tissues [2]. Although mitochondria are classically understood as organelles responsible for ATP production, there is increasing recognition that mitochondria are central in numerous additional cellular processes, including intracellular signaling, damage sensing and repair, oxygen and fuel sensing, cellular death, and inflammation. ROS are critical mediators of some of these aforementioned physiologic processes, especially when produced in low quantities. The electron transport chain comprises a series of proteins anchored to the inner mitochondrial membrane and is a key source of superoxide production. Through a sequence of redox reactions, this chain transfers electrons to liberate energy by producing a proton gradient which then generates ATP via ATP-synthase [50]. Under normal physiological conditions, this process functions smoothly, and healthy mitochondria are vital for the maintenance of vascular homeostasis. However, during episodes of oxidative stress, this finely tuned balance can be disrupted, leading to an escalation in the production of mitochondrial ROS [42]. Circular mitochondrial DNA is particularly susceptible to DNA damage by ROS and can be an indicator of ROS damage in chronic inflammatory diseases [50]. Oxidative damage further impedes the mitochondrion’s capacity to regulate the formation of ROS within the electron transport chain, creating a paradoxical positive feedback loop which results in the accumulation of damaged, dysfunctional mitochondria. Mitochondrial dysfunction and altered mitochondrial respiration have been linked to AAA formation [51]. Differential expression of genes associated with oxidative phosphorylation and mitochondrial function (e.g., *fibrillin-4*), have been identified in human AAA tissues, suggesting the association between the dysregulated production of mitochondrial ROS and aortic aneurysm formation [52]. Reduced expression of specific markers of mitochondrial biogenesis and vascular smooth muscle migration (e.g., PPARy coactivator-1-alpha) were identified in AAA tissues, further reinforcing the role of mitochondrial dysfunction in aortic aneurysm formation [53].

Oxidative stress can be a potent regulator of cellular signaling in many tissues, including the promotion of pro-inflammatory signaling, and the co-occurrence of oxidative stress and inflammation in human and pre-clinical models of AA disease give credence to the possibility that these processes interact to drive the pathophysiologic changes leading to AA formation. However, the specific association between the excessive generation of ROS and activation of the IL-1β pathway via the inflammasome pathway has some controversial linkages. Some studies in other diseases such as chronic granulomatous diseases (CGDs), which carry mutations in the *p47-phox* gene and are NADPH oxidase-deficient, are still able to activate IL-1 independent of ROS production; however, other inflammatory diseases are suggested to in part require ROS generation for IL-1 activation [54]. Further work is needed to provide a more comprehensive understanding of the interplay between oxidative stress and inflammatory signaling in AA disease.

## 5. Role of Endogenous Antioxidant Systems in Aortic Aneurysms

As previously discussed, oxidative stress is characterized by excessive levels of ROS relative to endogenous antioxidant defenses, and can be the result of the overproduction of ROS, deficiency of antioxidant systems or both. There exist several endogenous antioxidant systems that counteract the deleterious effects of ROS. Examples include Superoxide Dismutase (SOD), Glutathione Peroxidase (GPx), catalase, and Nuclear factor erythroid 2-related factor 2 (Nrf2). There are also several non-enzymatic antioxidant molecules including include iron and copper chelators, alpha-tocopherol (vitamin E), ascorbic acid (vitamin C), uric acid, melatonin, polyphenols, and polyamines (e.g., spermidine).

There is growing evidence that AAs are associated not only with increased abundance of pro-oxidant molecules but also with the disruption of endogenous antioxidant defense systems resulting in increased susceptibility to oxidative stress [55]. For example, plasma levels of alpha tocopherol (vitamin E) were observed to be lower in patients with AAA versus control patients [56]. Vitamin C levels have also been observed to be lower in aortic tissue from patients with AAA versus those without disease [57].

**Figure 2:**
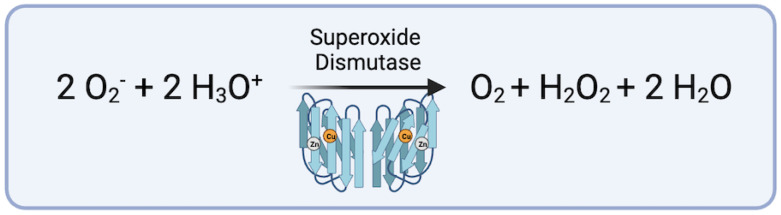
Conversion of superoxide anions via superoxide dismutase with cofactors Cu^2+^and Zn^2+^ (figure generated via biorender.com, accessed 1 November 2023).

Superoxide Dismutase (SOD) is an enzyme that facilitates the conversion of superoxide anions (O_2_^−^) into oxygen (O_2_) and hydrogen peroxide (H_2_O_2_), thus mitigating the detrimental effects of ROS (Figure 2) [58]. Elevated SOD levels have been observed in murine models of abdominal aortic aneurysms (AAA), suggesting a natural effort to counteract oxidative stress during aneurysm development [58,59]. Some studies have observed the decreased activity of CuZnSOD in aortic tissue from patients with AAA versus non-diseased control tissue [60,61]. The activity of SOD has been linked to the supplementation of riboflavin, vitamin B2, and will be discussed further in later sections [59].

**Figure 3:**
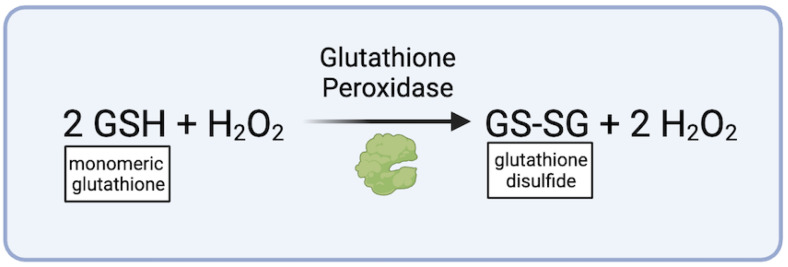
Conversion of hydrogen peroxide via Glutathione Peroxidase (figure generated via biorender.com, accessed 1 November 2023).

Glutathione Peroxidase (GPx), another enzyme, shields cell membranes from oxidative harm by reducing hydrogen peroxide, a byproduct of superoxide anion conversion, (H_2_O_2_) and lipid hydroperoxides into innocuous water (H_2_O) (Figure 3) [62]. Reduced GPx activity, resulting in increased oxidative burden and the activation of transforming growth factor-beta (TGF-β), has been linked to the formation of TAAs [63,64]. TGF- β has been established as a vital regulator of vascular function, with over-activation or over-inhibition being associated with AA formation [65].

**Figure 4:**
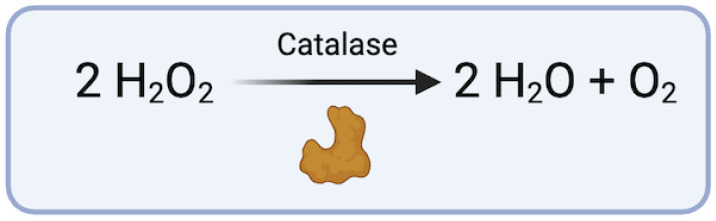
Conversion of hydrogen peroxide via catalase (figure generated via biorender.com, accessed 1 November 2023).

Similarly, the enzyme catalase, which converts hydrogen peroxide to water, has a vital role in the attenuation of MMPs (Figure 4). Reduced levels of catalase have been linked to the development of AAAs in murine models [66]. Given the association of the MMP degradation of aortic VSMC and the extracellular matrix, the upregulation of catalase in VSMCs within aortic tissues not only promotes the survival of these cells but also serves as a preventive measure against aneurysm formation by modulating MMPs [66,67]. Interestingly, the selective estrogen receptor modulator (SERM), tamoxifen, was found to exhibit vaso-protective effects in murine models through a five-fold increase in catalase mRNA production (*p* = 0.02) and an eight-fold increase in catalase protein production (*p* = 0.04) [67]. In this study, rats treated with tamoxifen had approximately 50% smaller AAA diameters when compared to control mice, supporting a role for catalase in the maintenance of oxidative balance and protection against AA formation [67].

**Figure 5:**
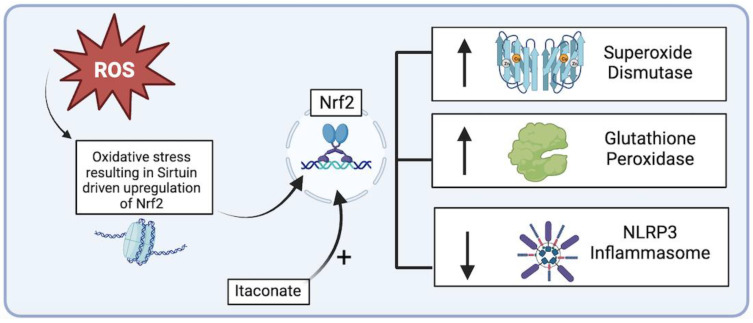
Sirtuin-driven Nrf2 upregulation of endogenous antioxidant systems and downregulation of NLRP3 inflammasome (figure generated via biorender.com, accessed 1 November 2023).

Important upstream regulators of endogenous antioxidant defense systems include Sirtuins, a family of histone deacetylases whose activity is important for regulating cellular functions including antioxidant defenses and inflammation [68]. Sirtuins help to regulate nuclear factor erythroid 2-related factor 2 (Nrf2), a master transcription factor which promotes transcription of antioxidant genes including SODs and catalase (Figure 5) [68,69]. The inhibition of nuclear Nrf2 has been associated with an increased risk of AAA development and rupture in murine models [70]. Additional studies in murine models found Itaconate, an anti-inflammatory mitochondrial metabolite produced by macrophages and monocytes, achieved protection against AAA formation via agonistic effects on the Nrf2 pathway and inhibition of the NLRP3 inflammasome [71]. A deficiency of Nrf2 resulted in increased inflammatory factor expression and AAA formation [71]. Upregulation of the protective Nrf2 antioxidant system and reduction in the NLRP3 inflammasome represents a novel and promising approach to aortic aneurysm treatment; however, clinical efficacy has yet to be established.

**Figure 6:**
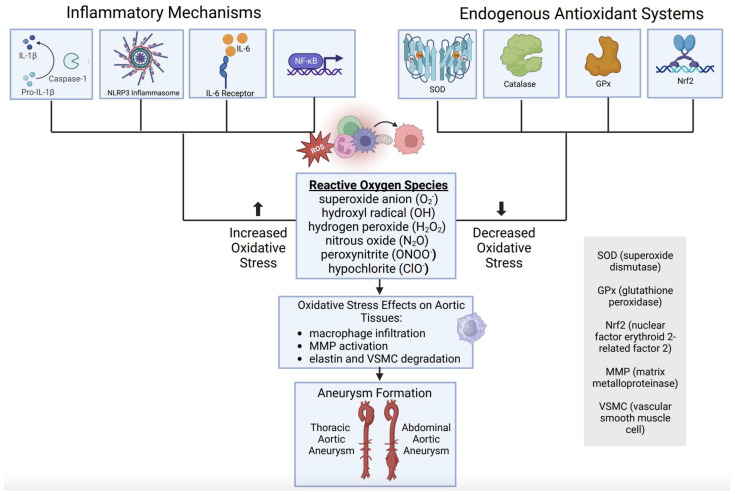
Oxidative stress imbalance role in aortic aneurysm formation. (figure generated via biorender.com, accessed 1 November 2023).

Together, available clinical evidence suggests that aortic aneurysm disease is associated with a relative deficiency of endogenous antioxidant defenses, which may contribute to a state of oxidative stress and subsequent activation of pathological signaling processes, including inflammation. This oxidative imbalance and inflammatory cascade result in changes in aortic tissue integrity, through macrophage infiltration, MMP activation, elastin degradation, and VSMC destruction in both thoracic and abdominal AAs (Figure 6).

## 6. Oxidative Stress as a Therapeutic Target in Aortic Aneurysms

Considering the role of oxidative stress in the pathophysiology of aortic aneurysms, there has been substantial interest in exploring the therapeutic potential of mitigating excessive ROS. Numerous preclinical and clinical investigations have delved into the feasibility of bolstering inherent antioxidant defenses with exogenous antioxidant compounds. However, only a limited number of studies have made connections between exogenous antioxidant supplementation and its potential to alleviate aortic aneurysm disease in humans, with limited evidence for its efficacy.

The potential role of various vitamins in preventing or treating aneurysms has been investigated, primarily due to their well-known potent antioxidant properties. Ascorbic acid (vitamin C), for instance, has demonstrated the ability to mitigate AAA development, preserving aortic elastin content, downregulating MMP-2/MMP-9 and IL-6 in murine models [72,73]. Prolonged treatment with vitamin C has also been shown to enhance nitric oxide synthase activity through the chemical stabilization of the cofactor tetrahydrobiopterin, observed in both murine models and human cultured human aortic tissues [74,75]. Despite these promising results, few clinical trials exploring the effect of ascorbic acid on human AA have been performed. Duffy et al. evaluated the antioxidant effects of vitamin C on ischemia–reperfusion injury during open AAA repairs; however, they found that parenteral ascorbic acid did not attenuate markers for systemic inflammation or endothelial damage [76].

Another vitamin explored for its impact on aneurysm formation is α-tocopherol, a form of vitamin E. α-tocopherol was found to reduce the formation of aortic aneurysms (resulting in a 24% reduction in maximal aortic diameter), decrease aortic rupture risk, lower aortic isoprostane content (a marker for oxidative stress), and reduce macrophage infiltration in ApoE-deficient murine models [77]. However, unlike vitamin C, vitamin E did not significantly affect MMP levels and had no impact on atherosclerosis [77]. Promising results from these studies prompted the evaluation of the clinical relevance and effectiveness of α-tocopherol and β-carotene (vitamin A) in humans. A randomized, double-blinded, placebo-controlled trial was conducted involving male smokers aged 50–69 years at the National Cancer Institute, with a mean follow-up period of 5.8 years. The results showed that vitamin E and β-carotene supplementation had no preventive effect on AAA formation or the risk of rupture [78].

Riboflavin, vitamin B2, was found to reduce AAA size and maintain elastin concentrations in aortic tissues through upregulation of endogenous SOD, in murine models [59]. Interestingly, in murine Marfan Syndrome models, a mixture of vitamin B6, B9, and B12 orally gavaged for 20 weeks was found to mitigate TAA formation and increase collagen deposition in the aortic media [79]. Overall, there remains a paucity of human trials involving antioxidant compounds demonstrating significant clinical effects on aortic aneurysms. However, therapeutic strategies which seek to augment endogenous antioxidant defense systems remain a promising though incompletely studied avenue of AA disease prevention.

Several preclinical studies in models of AAs suggest that therapeutic interventions which decrease oxidative stress are associated with the attenuation of vascular inflammation and aneurysm formation. Genetic overexpression of endogenous antioxidant catalase attenuated experimental aneurysm formation, accompanied by lower vascular inflammation [58]. In a preclinical study using a rat model of elastase-induced AAA, treatment with riboflavin increased endogenous SOD levels, and this was associated with the attenuation of AAA formation [59]. Melatonin treatment in a BAPN-induced mouse model of TAA increased aortic SIRT-1 abundance and activity, Nrf-2 abundance, and SOD activity and this increase in endogenous antioxidant defenses was associated with lower aortic markers of oxidative stress and, importantly, lower incidence of aneurysm formation and rupture [80]. In a murine model of angiotensin-II induced acute aortic dissection, treatment with ursodeoxycholic acid increased vascular Nrf2 expression, which was associated with the decreased expression of pro-oxidant NADPH oxidase and increased expression of antioxidant enzymes including CuZnSOD, MnSOD and catalase, and a resultant marked decrease in the incidence of aortic dissection [81].

Naturally occurring compounds with antioxidant properties also appear to attenuate vascular inflammation in preclinical studies. For instance, flavonoids, a group of phenolic substances found naturally in various fruits and vegetables, have been found to exhibit various beneficial biologic effects [82,83]. This group consists of nearly 3000 unique compounds, and have been linked to anti-inflammatory, antitumor, antioxidant, anti-platelet, and neuroprotective activities [82,83]. Diosmetin, a flavonoid derivative naturally occurring in citrus fruits, has demonstrated remarkable antioxidant capabilities in both in vivo and in vitro settings [84,85]. Believed to operate through the activation of the Nrf2 pathway, diosmetin has displayed anti-inflammatory properties, with indications of relief from cardiometabolic disorders, enhanced ventricular function, and coronary artery vasodilation in murine models [84,85]. Despite these promising discoveries, there is currently a lack of studies examining the impact of Diosmetin on aortic aneurysm formation, leaving space for future investigations in this domain. Many other natural compounds have been found to exhibit antioxidant properties yet lack significant clinical relevance.

Quercetin, a flavonoid found in onions, grapes, nuts, and broccoli has been explored in the realm of aortic aneurysmal disease given its potent antioxidant properties. Treatment of mice with quercetin was shown to prevent the onset of atherosclerosis, prevent macrophage infiltration of the aortic wall, suppress the formation of AAs, and prevent aortic dissection through reduction in the macrophage infiltration of aortic tissue, and cause a reduction in the activation of NF- Kβ and reduction in levels of MMP-2/MMP-9 [86,87]. Quercetin was found to exhibit endothelial cell-protective effects in cultured human umbilical vein endothelial cells, suggesting possibilities for human applicability [87]. Additional studies have replicated the protective effects of quercetin of AA formation, through the downregulation of cyclooxygenase-2 expression and VEGF signaling, and upregulation of various antioxidant mechanisms such as the SOD and caspase pathways [87,88]. To date, quercetin remains the only flavonoid with evidence suggesting direct benefit in AA prevention and treatment.

Overall, there are limited but compelling preclinical data to suggest that therapeutic strategies to augment endogenous antioxidant defense systems may hold promise for attenuating aortic aneurysm development (Table 1). Given limited translational investigation, future work is required to establish the importance of antioxidant systems in the prevention or augmentation of AAs.

## 7. Conclusions

AAs are a chronic vascular disease that currently have no medical treatment therapy; therefore, identification of non-invasive medical therapies to attenuate or slow aneurysm growth is a valuable biomedical research priority with potential for significant clinical impact. The pathophysiology of aneurysm formation is complex and remains poorly understood, but current evidence suggests that oxidative stress and inflammation are signaling processes which interact to drive detrimental changes to the vascular wall. Oxidative stress results both from the excess abundance of ROS as well as the deficiency of endogenous antioxidant and scavenging mechanisms. There is a body of preclinical work suggesting the promise of therapeutic interventions which target excessive ROS and/or augment endogenous antioxidant systems for attenuating AA development; however, many of these therapeutic strategies have failed to show clinical benefit in humans despite success in preclinical disease models. Therefore, as future work develops novel targets, there is the need for the greater translational efficacy of these targets for human disease.

## Figures and Tables

**Table 1:** Summary table of exogenous antioxidants and their effect on AA.

Exogenous Antioxidants	In Vivo/In Vitro Effects on Aortic Aneurysms (AA) [Murine Models]	References
**riboflavin (vit. B2)**	Reduction of Abdominal AA maximal diameter Preservation of aortic elastin concentration Upregulation of endogenous Superoxide Dismutase Increased collagen deposition in Thoracic AA	[59,79]
**Itaconic acid (itaconate)**	Attenuation of Abdominal AA formation through Nuclear factor erythroid 2-related factor 2 (Nrf2) upregulation	[71]
**Ascorbic acid (vit. C)**	Preservation of aortic elastin concentration Downregulation of matrix metalloproteinase (MMP-2, MMP-9), and Interleukin-6	[72,73,74,75]
**α-** **tocopherol (vit. E)**	Reduction of AA maximal diameter Decrease aortic rupture rate Lowers markers of aortic oxidative stress	[77,78]
**Melatonin**	Lowers incidence of Thoracic AA formation and rupture Increased endogenous Superoxide Dismutase Lowers markers of oxidative stress in aortic tissues	[80]
**Ursodeoxycholic acid**	Reduction of acute aortic dissection Decreased expression of NADPH oxidase Increased endogenous Superoxide Dismutase and catalase	[81]
**Diosmetin**	Endogenous Nrf2 upregulation Relief of cardiometabolic disorders Enhanced ventricular function Coronary artery vasodilation	[84,85]
**Quercetin**	Downregulation cyclooxygenase-2 and VEGF Upregulation of Superoxide Dismutase and caspase Endothelial cell protection *	[86,87,88]

* studies performed in cultured human cells.

## Abbreviations

AA	aortic aneurysms
AAA	abdominal aortic aneurysm
aAA	ascending aortic aneurysm
ApoE	apolipoprotein E
dTAA	descending thoracic aortic aneurysm
BAPN	B-aminopropionitrile
CD4	cluster of differentiation 4
CuZnSOD	copper zinc superoxide dismutase
dTAA	descending thoracic aortic aneurysm
ECM	extracellular matrix
eNOS	endothelial nitric oxide synthase
GPx	glutathione peroxidase
IL-6	interleukin 6
IL-17	interleukin 17
IL-23	interleukin 23
IL-1α	interleukin-1 alpha
IL-1β	interleukin-1 beta
IL-1R1	interleukin-1 receptor 1
IL-R1	interleukin receptor 1
iNOS	inducible nitric oxide synthase
IFN𝛾	interferon gamma
MCP1	monocyte chemoattractant protein 1
MMP-2	matrix metalloproteinase 2
MMP-9	matrix metalloproteinase 9
MnSOD	manganese superoxide dismutase
NLRP3	NLR family pyrin domain containing 3
NF-Kβ	nuclear factor kappa beta
NRF2	Nuclear factor erythroid 2-related factor 2
PPAR𝛾	peroxisome proliferator-activated receptor gamma
ROS	reactive oxygen species
SOD	superoxide dismutase
TNF-α	tumor necrosis factor alpha
TGF-β	transforming growth factor beta
VEGF	vascular endothelial growth factor
VSMC	vascular smooth muscle cell
